# Motivational consequences influence obstacle crossing strategies

**DOI:** 10.3389/fnhum.2026.1756616

**Published:** 2026-03-31

**Authors:** Abigail C. Schmitt, Ashlyn M. Jendro, Isabella Champenois, Tiphanie E. Raffegeau

**Affiliations:** 1 Department of Health, Human Performance, and Recreation, University of Arkansas, Fayetteville, AR, United States; 2 School of Kinesiology, George Mason University, Manassas, VA, United States

**Keywords:** consequences, deception, gait, motivation, obstacle crossing

## Abstract

Young adults are likely to experience fewer physical consequences and less psychological stress associated with falls, despite frequent tripping and falling. Negative consequences associated with a task can stimulate anxiety, altering the obstacle crossing approach. This study employed motivational consequences of time and money to investigate how simulated anxiety alters obstacle crossing in young adults. We hypothesized negative motivational consequences would elicit more successful and cautious obstacle crossings. Sixty young adults, randomized into control and consequence groups, crossed obstacles while three-dimensional motion capture measured trajectories and joint kinematics during obstacle avoidance. The control group was 3.44 times more likely to contact an obstacle than the consequence group, demonstrating the consequences resulted in more successful crossings. Further, the consequence group adopted a more cautious avoidance strategy, exhibited by greater foot clearance and greater crossing step lengths, despite the consistent task constraints. These findings illustrate the importance of motivation and perceived consequences in obstacle crossing.

## Introduction

Approximately, 1 out of every 4 falls that occur during walking are on uneven terrain (Milat et al., 2011; Timsina et al., 2017). Additionally, an exceptionally large number of injuries and hospitalizations are due to falls when attempting to navigate an obstacle (Hahn and Chou, 2004; Timsina et al., 2017). Falls from unsuccessful obstacle crossing attempts can result in both physical and psychological consequences, including a fear of falling and post-traumatic stress (Jayasinghe et al., 2014). Despite 60% of young adults tripping and nearly 50% of young adults falling (Cho et al., 2021), concern regarding falls as a health risk to young adults is uncommon. Further, young adults exhibit higher rates of obstacle contacts than older adults in laboratory studies, although older adults may be more likely to sustain an injury after an obstacle contact (Muir et al., 2020).

Obstacle crossing research typically describes common strategies healthy and clinical populations employ when crossing obstacles. For practical purposes, investigations are often conducted in laboratory environments that are relatively safe and lack consequences that would be associated with obstacle crossing in the real world. Successful stationary obstacle avoidance requires motor planning and gait adjustments during the approach steps, involving visual integration of the obstacle location, dimensions, and approach speed to plan subsequent foot placement, limb elevation, and step length modulation (Mohagheghi et al., 2004; Patla and Greig, 2006; LoJacono et al., 2018). Obstacle avoidance strategies can be quantified via vertical limb clearances, horizontal limb clearances (e.g., approach and landing distances), and joint angles using motion capture to measure movement.

Laboratory-based assessments of obstacle crossing often necessarily lack the natural physical consequences (i.e., tripping or falling) of unsuccessful crossings that are present in the real world. Rather than elicit naturalistic trips or falls, and in an effort to balance participant safety and natural behavior, risk-averse methodological choices must be made in laboratory settings (McFadyen and Carnahan, 1997; Pijnappels et al., 2001). For example, the decision to use a safety harness may alleviate potential injuries from falls but could alter natural gait mechanics and neuromechanical task constraints (Decker et al., 2012; Augenstein et al., 2025). Further, the type of obstacle may be intentionally designed to mitigate injury in the case of a contact, at the expense of realism and authenticity (Jendro et al., 2025). Considering most young adults are not at risk of falling from a trip or an unsuccessful obstacle crossing because of sufficient sensorimotor skills (Brown et al., 2005; Cho et al., 2021), physical consequences from a trip or fall may not be as meaningful/salient to young people. As such, data from young adult obstacle crossing studies can be difficult to interpret and challenging to translate to older adults. Specifically, it remains unclear if young adults cross obstacles carelessly since they do not bear the same potential consequences as older adults, or more impaired populations, or if young adults prioritize crossing efficiency over safety.

Evidence suggests the presentation of negative consequences stimulates anxiety in young adults, imposing additional cognitive demands to prevent the consequence, which can affect the way a task is performed (DeCouto et al., 2021). To simulate anxiety that may be associated with an unsuccessful obstacle crossing for older adults, this study implemented two highly motivational consequences for young adults to provoke anxiety: time (Misra and Mckean, 2000), and money (Pessiglione et al., 2007; Scott-Clayton, 2011; Thibault Landry et al., 2020). Thus, we sought to assess how manipulating negative motivational consequences alters measures of obstacle crossing in young adults, across different real-world obstacles known to prompt different crossing strategies (Jendro et al., 2025). We implemented motivational consequences in the form of a deception regarding loss of time and money to evaluate how provoked anxiety modifies measures of obstacle avoidance. We hypothesized that young adults who were given negative motivational consequences would cross obstacles with a more cautious strategy and have more successful obstacle crossings as compared to a control group who received no motivational consequences or specific directions.

## Methods

A portion of these data (the control group results) have been previously published (Jendro et al., 2025). The current analysis and experiment test a unique and novel hypothesis using a comparison group to test motivational consequences.

### Participants

Sixty young adults were randomly divided into two groups: a control group (10 men, age, 23 ± 4 years, height, 1.66 ± 0.10 m; mass: 69.7 ± 16.5 kg; overground, unobstructed preferred walking speed: 1.25 ± 0.14 m/s) and an experimental group (12 men, age, 21 ± 1 years, height, 1.70 ± 0.09 m; mass: 69.4 ± 11.1 kg; overground, unobstructed preferred walking speed: 1.26 ± 0.13 m/s). Participants reported no diagnostic history of neurological or orthopedic problems that could impair walking ability or general mobility and could walk unassisted for >15 min at a time. Written informed consent was obtained prior to participation, and the study was approved by the University’s Institutional Review Board (protocol #2105335358).

### Procedures

Participants followed procedures previously described in Jendro et al. (2025) and described briefly herein. Participants wore the Plug-in-Gait full-body marker set (Vicon Motion Systems, Oxford, UK) and walked at their preferred, comfortable pace under 6 conditions while barefoot: an unobstructed walking condition, and five obstructed walking conditions. The order of the five obstructed walking conditions was randomized and included: (1) a branch (170 mm tall, 25 mm deep), (2) a parking curb (125 mm tall, 215 mm deep), (3) a dowel rod (150 mm tall, 25 mm deep), and (4) a rope (300 mm tall, 50 mm deep). The fifth obstructed walking condition, the puddle condition, was excluded from this analysis because of its similarities to unobstructed overground walking from previous analyses (Jendro et al., 2025). Each condition included 10 overground walking trials along an 8-meter walkway. No practice or familiarization trials were given. Obstacles were positioned within full view of participants. Three-dimensional marker trajectories for both the participant and the obstacle were simultaneously captured using a 16-camera Vicon motion capture system. An example of the experimental setup and images of the obstacles can be found at Jendro et al. (2025).

For the obstacle crossing trials, participants were asked to “…walk across the walkway at a comfortable pace…stepping over the [obstacle] in your path along the way”. Participants in the control group were given no additional instructions. Experimental group participants were informed that they would be paid $15 if they successfully crossed all obstacles without bumping, touching, contacting, or altering the obstacle in any way and, if they did contact the obstacle, they would forfeit the money they would have earned for that obstacle and would have to recollect all trials of the contacted obstacle at another time (full script in Appendix A). The experimental group was reminded of the consequences before each obstacle condition began. The experimental group was the only group to receive financial compensation. After all study procedures were completed, experimental participants were unblinded to the deception and provided an explanation (see Appendix A). All participants in the experimental group received compensation and did not have to recollect trials regardless of if they contacted the obstacle.

### Data analysis

Trajectory data was reconstructed, gap-filled, and filtered using a low-pass, fourth order, zero-lag Butterworth filter with a cut-off frequency set to 6 Hz in Nexus (Vicon Motion Systems, Oxford, UK), prior to the Plug-in-Gait model calculations. Exported data was processed using custom MATLAB (MathWorks, Natick, MA, USA) algorithms to extract the variables of interest.

Measures of horizontal and vertical foot clearance were obtained to measure obstacle crossing strategy and included approach and landing distances for both the lead and trail limbs and vertical foot clearance for both the lead and trail limbs. Approach distance was defined as the horizontal distance between the toe marker of the respective limb and the obstacle prior to the crossing step and provides insight into adaptive step-length modulation strategies. Landing distance was defined as the horizontal distance between the heel marker of the respective limb and the obstacle after crossing. Vertical foot clearance was defined as the minimum vertical distance between either the toe (i.e., second metatarsal) or heel (i.e., posterior calcaneus) marker of the respective limb, whichever was smaller, and the obstacle when the marker was directly above the obstacle. Crossing step length was defined as the difference between the positions of the heel marker of the trailing limb at approach foot-strike and the heel marker of the leading limb at landing foot-strike. Gait velocity was calculated as the center of mass velocity for both the middle 4 m of the walkway and for the crossing phase. Peak lower extremity joint angles were extracted during the crossing step, when the foot was over the obstacle.

All variables of interest were extracted from each trial and averaged within obstacle conditions for every participant. Unsuccessful trials, where participants contacted the obstacles, and the following trial were removed from the analysis.

### Statistical analysis

To compare crossing strategies between groups, we decided *a priori* to examine the main effect of motivational consequences (group) and the interaction between the group and obstacle, but not the main effect of obstacle nor the *post-hoc* pairwise interactions between every combination of group and obstacle. Data from the control group examining the main effect of obstacle can be found at Jendro et al. (2025). A series of linear mixed-effects regression models (*lmer*) was used to perform 2 × 4 repeated measures analysis of variance (ANOVA) tests to compare the dependent variables between groups and to analyze the relationship between group and obstacle. If the ANOVA for the group × obstacle interaction resulted in significant differences (*p* < 0.05), *post-hoc* comparisons (*emmeans*) were adjusted using False Discovery Rate (FDR) corrections (*α* = 0.05, number of comparisons = 10) (Benjamini and Hochberg, 1995; Glickman et al., 2014). Cohen’s *d* was used to characterize the magnitude of the effect between pairwise comparisons (*eff_size* with summed variance components). Effect size was interpreted as small (0.2 ≤ *d* < 0.5), medium (0.5 ≤ *d* < 0.8) or large (*d* ≥ 0.8) (Cohen, 1988).

To model the probability of obstacle contacts between the control group and the consequence group and to account for most participants with zero obstacle contacts, a binomial logistic regression model, with outcome modeled as the number of success and failures within each participant (i.e., modeled as a probability contact per participant), was utilized. As a measure of effect size, the odds ratio was calculated (i.e., control group divided by consequence group) using the estimated marginal means (*emmeans*). Significance level was set at *p* < 0.05. All statistical analyses were completed using R (v.4.4.0 “Puppy Cup”) and RStudio (v.2024.04.1 + 748 “Chocolate Cosmos”) (R Core Team, 2024).

## Results

There were 17 unsuccessful trials among participants in the control group (2 curb, 15 rope) and 5 unsuccessful trials among participants in the consequence group (1 curb, 4 rope, Table 1). The probability of contacting the obstacle was 1.38% for the control group and 0.41% for the consequence group (Table 1). Participants in the control group were 3.44 times the odds to contact an obstacle (*p* = 0.016).

**Table 1:** Number of trials and hits for each obstacle by group.

Group	Curb		Dowel		Rope		Branch		Total	
	Trials	Hits	Trials	Hits	Trials	Hits	Trials	Hits	Trials	Hits
Control	303	2 (0.7%)	305	0 (0%)	319	15 (4.7%)	304	0 (0%)	1,231	17 (1.4%)
Consequences	307	1 (0.3%)	308	0 (0%)	310	4 (1.3%)	308	0 (0%)	1,233	5 (0.4%)

Hits were defined as trials where any form of contact between the participant’s foot and the obstacle occurred.

### Horizontal clearance variables

Approach distance of the trailing limb differed significantly between groups [*F*(1,60) = 11.16, *p* = 0.001, *d* = 0.78], with the consequence group exhibiting greater approach distances of the trailing limb, but no significant group × obstacle interaction was detected [*F*(3,180) = 1.15, *p* = 0.330]. Landing distance of the trailing limb displayed a significant group × obstacle interaction [*F*(3,180) = 3.03, *p* = 0.031], which was driven by pairwise differences between the obstacles, within the consequence group, rather than group differences (branch *p* = 0.905, *d* = 0.46 curb *p* = 0.701, *d* = 0.57, dowel *p* = 0.873, *d* = 0.40, rope *p* = 0.209, *d* = 0.75). Specifically, the curb obstacle elicited a shorter landing distance of the trailing limb than any of the other obstacles in the consequence group (curb-branch *p* = 0.001, *d* = 0.94, curb-dowel *p* = 0.001, *d* = 0.92, curb-rope *p* < 0.001, *d* = 0.77), while none of the other obstacles differed from each other (branch-dowel *p* > 0.999, *d* = 0.02, branch-rope *p* > 0.999, *d* = 0.16, dowel-rope *p* = 0.936, *d* = 0.14). There were no significant group differences or group × obstacle interactions for either the approach distance of the leading limb or landing distance of the leading limb (Table 2, all *p* > 0.05).

**Table 2:** Horizontal clearance measures, vertical clearance measures, and spatiotemporal variables of interest across groups for each obstacle (mean ± standard deviation).

Group	Obstacle	Lead approach distance (mm)	Trail approach distance (mm)	Lead landing distance (mm)	Trail landing distance (mm)	Lead vertical clearance (mm)	Trail vertical clearance (mm)	Crossing step length (mm)	COM velocity (mm/s)	COM velocity during crossing (mm/s)
Control	Branch	907 ± 100	256 ± 60	239 ± 50	882 ± 85	122 ± 36	156 ± 44	701 ± 70	1,161 ± 107	1,091 ± 111
Consequence		966 ± 116	**308 ± 58**	240 ± 54	**923 ± 113** ^c^	**162 ± 48** ^c,d,r^	**226 ± 61**	**753 ± 77**	1,168 ± 126	1,079 ± 140
Control	Curb	823 ± 94	182 ± 53	145 ± 39	787 ± 74	96 ± 32	108 ± 26	742 ± 67	1,188 ± 119	1,143 ± 118
Consequence		866 ± 105	**216 ± 40**	156 ± 37	**839 ± 89** ^b,d,r^	124 ± 31^b,d,r^	**160 ± 43**	**789 ± 64**	1,195 ± 117	1,147 ± 130
Control	Dowel	908 ± 104	259 ± 58	241 ± 50	886 ± 82	147 ± 42	191 ± 44	709 ± 69	1,162 ± 115	1,096 ± 122
Consequence		967 ± 134	**304 ± 67**	235 ± 52	**922 ± 109** ^c^	**186 ± 43** ^b,c^	**257 ± 62**	**747 ± 78**	1,152 ± 130	1,065 ± 149
Control	Rope	914 ± 101	268 ± 54	198 ± 40	840 ± 74	134 ± 44	215 ± 58	719 ± 75	1,103 ± 111	981 ± 125
Consequence		956 ± 120	**307 ± 53**	209 ± 47	**908 ± 101** ^c^	**192 ± 44** ^b,c^	**294 ± 60**	**768 ± 68**	1,093 ± 125	971 ± 153

Group differences (consequence vs. control) are indicated with bold values and pairwise differences among obstacles are denoted with a superscript letter for the obstacle (i.e., b, branch, c, curb, d, dowel, r, rope).

### Vertical clearance variables

**Figure 1:**
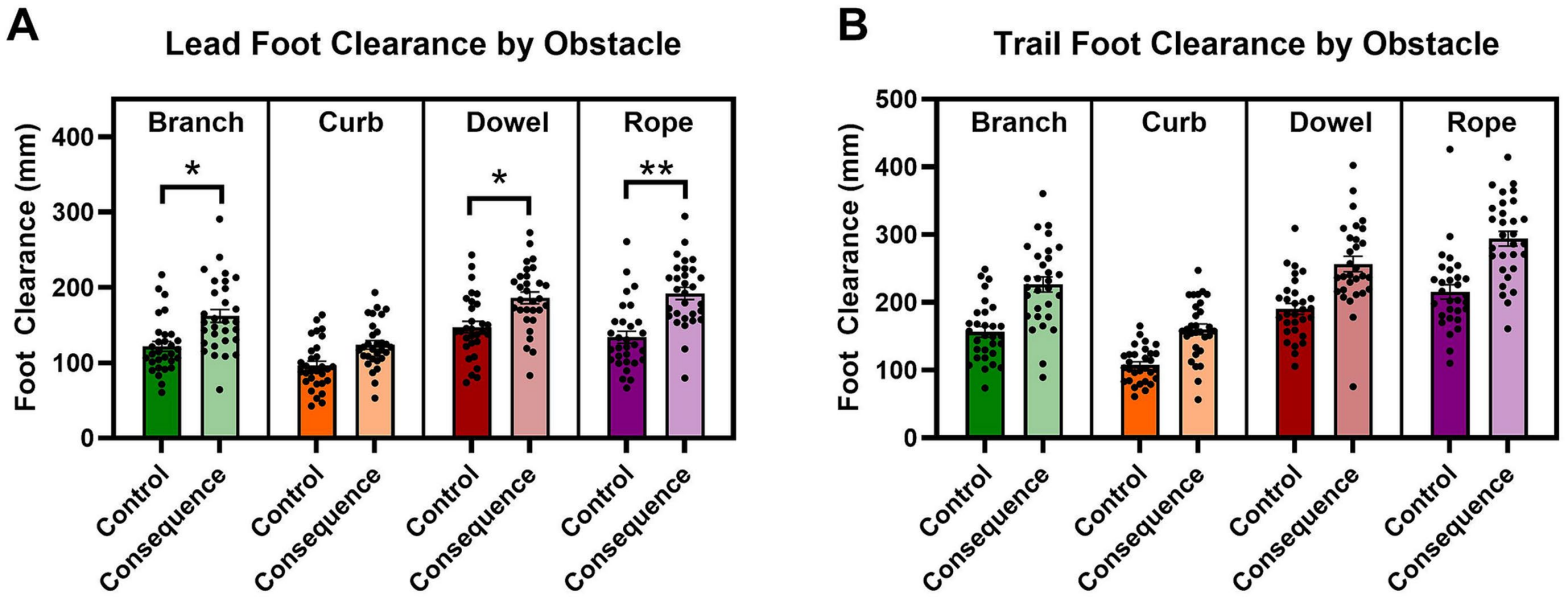
Mean ± standard error of the mean (error bars) with individual data points of the lead foot vertical clearance [left panel, **(A)**] and the trail foot vertical clearance [right panel, **(B)**]. A group × obstacle interaction revealed significant differences in lead foot vertical clearance between the control and consequence groups for the branch (*p* = 0.006), the dowel (*p* = 0.008), and the rope (*p* < 0.001) and a group main effect revealed the consequence group exhibited significantly more trail foot vertical clearance than the control group, across all obstacles (*p* < 0.001).

Lead foot vertical clearance exhibited a significant group × obstacle interaction [F(3,180) = 5.65, *p =* 0.001], which revealed the consequence group exhibited greater clearance than the control group for all obstacles except the curb (curb: *p* = 0.153, *d* = 0.70, all others: *p* ≤ 0.008, *d* range = 0.98–1.46, Figure 1A). Within the consequence group, clearance differed between all combinations of obstacles except the dowel and the rope (*p* = 0.954, *d* = 0.15, all others: *p* ≤ 0.001, *d* range = 0.60–1.72). Collectively, these data indicate the consequence group exhibited greater clearance over the obstacles than the control group, and the consequence group displayed the smallest foot clearance over the curb, followed by the branch, then followed by the dowel and rope. Trail foot vertical clearance differed significantly between groups [*F*(1,60) = 38.09, *p <* 0.001, *d* = 1.33], with the consequence group exhibiting greater vertical clearance above the obstacles than the control group (Table 2, Figure 1B).

### Spatiotemporal variables

Crossing step length differed significantly between groups [*F*(1,60) = 7.29, *p* = 0.009, *d* = 0.67], with the consequence group taking longer steps than the control group, regardless of obstacle. There were no significant group differences or group × obstacle interactions between groups in gait velocity, for either the 4 m gait speed or the crossing speed (Table 2).

### Kinematic variables

Among the lower extremity joint angles, only the trailing limb peak knee angle [*F*(1,60) = 12.54, *p* < 0.001, *d* = 0.76] and the leading limb peak hip angle [*F*(1,60) = 4.46, *p* = 0.039, *d* = 0.53] differed between groups. The consequence group crossed the obstacles with greater peak flexion for both the trailing limb knee and the leading limb hip (Table 3). No other lower extremity peak joint angles differed between groups or exhibited significant interactions between group and obstacle.

**Table 3:** Peak lower extremity joint angles over each obstacle across groups for each obstacle (mean ± standard deviation).

Group	Obstacle	Lead ankle angle	Trail ankle angle	Lead knee angle	Trail knee angle	Lead hip angle	Trail hip angle
Control	Branch	5.1 ± 4.4	−4 ± 8.7	90 ± 10.2	105.9 ± 8.5	66.4 ± 8.8	29.9 ± 7.3
Consequence		3.3 ± 5.9	−1.9 ± 8.3	91.9 ± 11.4	**113 ± 9.7**	**71.4 ± 10.7**	32.6 ± 9.2
Control	Curb	6.6 ± 3.9	−5.8 ± 8.4	85.4 ± 10.7	99.2 ± 6.7	62.3 ± 8	31.2 ± 7
Consequence		5.7 ± 5.6	−2.9 ± 7.2	89.1 ± 10	**104.4 ± 8.7**	**66.7 ± 9.9**	33.8 ± 8.4
Control	Dowel	6.4 ± 5.1	−4.8 ± 10	91.9 ± 10	108 ± 6.7	67.6 ± 8.5	29.8 ± 7
Consequence		4.7 ± 5.5	−2.2 ± 8.1	94.4 ± 10.2	**115.4 ± 9.6**	**72.5 ± 10.9**	33.5 ± 9.4
Control	Rope	8.6 ± 5	7.2 ± 9.3	108.2 ± 9.2	128 ± 7.5	79.8 ± 9.4	34.1 ± 8.4
Consequence		7.6 ± 5.9	8.4 ± 7.7	113.7 ± 12.8	**132.8 ± 7.8**	**85.7 ± 10.8**	35.9 ± 13.3

Group differences (consequence vs. control) are indicated with bold values.

## Discussion

The purpose of this study was to assess the effects of motivational consequences on measures of obstacle crossing in young adults. We hypothesized that motivational consequences would result in more cautious crossing strategies and more successful obstacle crossings. Our hypotheses were largely supported, the consequence group did exhibit a more cautious crossing strategy, illustrated by greater foot clearance measures. Further, the consequence group had a greater proportion of successful crossings than the control group, illustrating an anxiety-like response provoked by the motivational consequences served to promote a safer, more successful crossing strategy. Collectively, these results highlight that motivational consequences alter the way young adults negotiate obstacles, leading to a more cautious strategy for obstacle avoidance. We posit this strategy was adopted to avoid the negative consequence associated with contacting the obstacle.

Individuals in the consequence group displayed higher lead foot clearances, using increased lead limb hip flexion to raise the leading foot up and over the obstacle. Similarly, the consequences elicited increased trail limb knee flexion, providing trail foot elevation which resulted in greater trail foot clearance. Greater vertical foot clearance illustrates the shift towards a more conservative, cautious crossing strategy, which acts to increase the margin of safety and reduce the probability of tripping for the consequence group. Trail limb foot clearance is typically larger than lead limb foot clearance during successful obstacle crossings (Sparrow et al., 1996), due to the lack of visual input to provide real-time feedback and error reduction to the trailing limb during crossing (Patla, 1998; Rietdyk and Rhea, 2011). The consequence group demonstrated more caution with trail foot crossings, with no available visual feedback, across all obstacles, supporting that they avoided the obstacles more intentionally than control participants regardless of the type of obstacle and its dimensions.

Motivational consequences prompted participants to place the trailing foot further from the obstacles prior to crossing, supported by greater horizontal clearance measures (i.e., approach and landing distance of the trail limb), rather than a combination of longer lead limb landing distance and approach distance. The lack of differences in the lead foot landing distance highlights that the consequence group took a larger crossing step to achieve successful crossings than the control group. Additionally, the consistency of the landing distance of the lead foot may be more indicative of task constraints than strategy; the obstacles were consistent between groups and only successful crossings were analyzed. It may be that, after a successful crossing, initial placement of the lead foot is less meaningful, as it relates to tripping, than the approach or subsequent recovery steps. The initial foot-strike serves to provide a stable base, and its position determines the size of the base of support during the crossing step. Subsequent steps function to re-establish consistent walking patterns and maintain forward progress.

Despite our consequence group taking a longer crossing step, they maintained similar gait speeds as the control group. Individuals in the consequence group compensated by taking less time to place their steps, preserving speed while crossing all obstacles- regardless of the dimensions. Therefore, the consequence group demonstrated an ability to violate Fitt’s Law by compensating for more cautious crossing strategies by increasing accuracy without compromising speed. The present results likely represent an ideal strategy for obstacle avoidance in a healthy population. In older adults, the tradeoff between increasing obstacle clearance and slower speeds is consistent with Fitt’s law (Muir et al., 2015).

Although the dimensions among our real-world obstacles varied, the physical requirements/task constraints did not differ between the consequence and control groups. This is particularly interesting considering the threat implied by the obstacles should communicate a need for caution, which should be equivalent in both groups. However, our experiment- which did not add any actual physical risk- made the consequence group more cautious when avoiding the obstacles. Indeed, the control group contacted the obstacle 3.4 times more than the consequence group. The differences in crossing strategy seem to arise from motivation or performance pressure that elevates the level of caution the obstacle necessitates beyond its physical dimensions and actual risk of tripping. The probability of falling is a perceptual risk variable that influences stability and foot placement in healthy adults (Seddighi et al., 2025), which may help explain the differences observed in horizontal clearance and crossing step length. Our results suggest that, regardless of the characteristics of the obstacle, the level of caution is affected by motivation and external motivation may serve to augment caution.

Although this study employed a psychological approach to motivating consequences relevant to young adults—time and money—these findings can be extrapolated to provide insights into obstacle crossing in older adults. Older adults often adopt more conservative strategies to ensure successful obstacle crossing, like higher clearances and slower speeds than young adults, likely related to the higher risk of injury resulting from a fall (Chen et al., 1991; Scheffer et al., 2008). Additionally, older adults can experience psychological trauma after injurious falls (Jayasinghe et al., 2014) and a fear of future falls even without experiencing a fall (Hadjistavropoulos et al., 2011), which may lead to increased caution and a more conservative approach for obstacle crossing. Similarly, it appears that young adults presented with motivational consequences also adopt a conservative crossing strategy, despite a lower risk of physical or psychological consequences from falling compared to older adults. We recognize the underlying cause of the anxiety experienced by older adults due to fear of falling and the young adults’ motivational consequences presented in this experiment are not analogous. However, as our consequence group adopted a strategy often associated with older adults, the motivational consequence model appears to increase performance pressure and evoke an anxiety-like response, leading to an altered obstacle avoidance approach.

Our study did not incorporate any measures of anxiety, either self-report or physiological response measures, but rather relied on observations during the data collection. This allowed us to preserve the deception paradigm, although future research could integrate state and trait anxiety measures to better understand the influence of the motivational consequences, leading to a better understanding of how anxiety impacts obstacle negotiation strategies. Further, it should be noted that our laboratory-based tasks may not fully reflect everyday obstacle negotiation or elicit the cautiousness associated with fear-of-falling related anxiety. Our study demonstrates that motivation plays a role in the selection of obstacle negotiation strategies, which could have implications for clinical practice. Future research should explore the influence of motivation on real-world obstacle negotiation and expand on these findings to include clinical populations.

In conclusion, our results demonstrate that motivational consequences promote a more cautious and successful obstacle crossing strategy in young adults. These results expand our understanding of how perceived consequences influence obstacle avoidance strategies, despite consistent physical task constraints. Better understanding motivation for cautious gait strategies could help improve rehabilitation techniques and fall recovery training by leveraging motivation to promote safe obstacle crossing strategies.

## Data Availability

The raw data supporting the conclusions of this article will be made available by the authors, without undue reservation.

## Appendix A: study scripts

### Script to be read during the study

*“You will complete a minimum of 10 trials for each of the five different obstacles. You will have the opportunity to earn $15 at the end of the experiment if the following conditions are met. You must successfully cross each obstacle without bumping the obstacle, altering the obstacle in any way, touching the obstacle, tripping over the obstacle, slipping, or falling. If this were to occur during one of your trials, you will lose the money you could have possibly earned for that obstacle (which is 3 dollars per obstacle). Additionally, you will have the option to stay at the end of the visit or reschedule so we can recollect the trials of the obstacle you hit to make sure we get all the data we need. Again, if you complete all trials successfully, you will receive $15 dollars in full.”*

### Script to be read after the study

*“Now that you have completed the minimum of 10 trials for each of the five different obstacles, we can tell you a bit more about the study. In addition to looking at how you cross the obstacles, we were interested in knowing if you would cross them differently in the presence of a consequence. We think that motivating consequences may impact how people cross obstacles. We told you that you would have the opportunity to earn $15 at the end of the experiment only if you completed all of the obstacle crossings successfully, but we are actually giving you $15 no matter what. You also do not need to repeat any trials that were unsuccessful, that was another thing we said to motivate you to do well. We are sorry we had to deceive you, but we wanted to know if your motivation changed your behavior. Thank you for participating in the study and helping us better understand how people cross obstacles.”*

### We have two last things

1. *If you happen to know anyone else participating in the study, please do not tell them anything about the study.*
2. *Do you feel like the $15 and the potential extra time were good motivators for you to do well? If not, what do you feel like would have convinced you to try harder?”*
